# Learning science alongside peers with intellectual and developmental disabilities

**DOI:** 10.1371/journal.pbio.3002147

**Published:** 2023-06-13

**Authors:** Kaelin N. Rubenzer, Jonathan T. Pierce

**Affiliations:** Department of Neuroscience, The Center for Learning and Memory, Waggoner Center for Alcohol and Addiction Research, Institute of Neuroscience, University of Texas at Austin, Austin, Texas, United States of America

## Abstract

Science should be a subject that can be enjoyed by students of all abilities. This Community Page describes a program that enables college students to learn advanced science topics alongside adults with intellectual and developmental disabilities and explains how similar programs can be implemented.

## Teaser

“Lifelong Learning with Friends” provides diversity training to college students by having them learn science alongside adults with intellectual and developmental disabilities (IDDs). Volunteers showed increased interest in IDD-focused research, social interaction, and advocacy.

## Connecting college students to peers with disabilities

There is a rising demand to expand diversity training for future scientists, physicians, and educators to include real-world experiences [1]. Diversity training often focuses on critical issues like race, gender, and socioeconomic status, but overlooks intellectual and developmental disabilities (IDDs) [2–4]. The Special Olympics has offered valuable diversity-equity-inclusion (DEI) experiences to learn about people with IDDs through “Unified Sports”and “Best Buddies” [5–7], but these programs still do not necessarily demonstrate the level of what adults with IDDs are capable of learning. In 2010, we sought to combine elements of diversity training with experiential learning in a novel program, Lifelong Learning with Friends (LLWF), to connect science, technology, engineering, and math (STEM) and premedical college students to peers with IDDs. We hypothesized that having college students learn sophisticated subjects, including science, alongside adults with IDDs would increase college students’ expectations and interest in IDD-focused research, education, social interaction, and advocacy.

**Fig 1:**
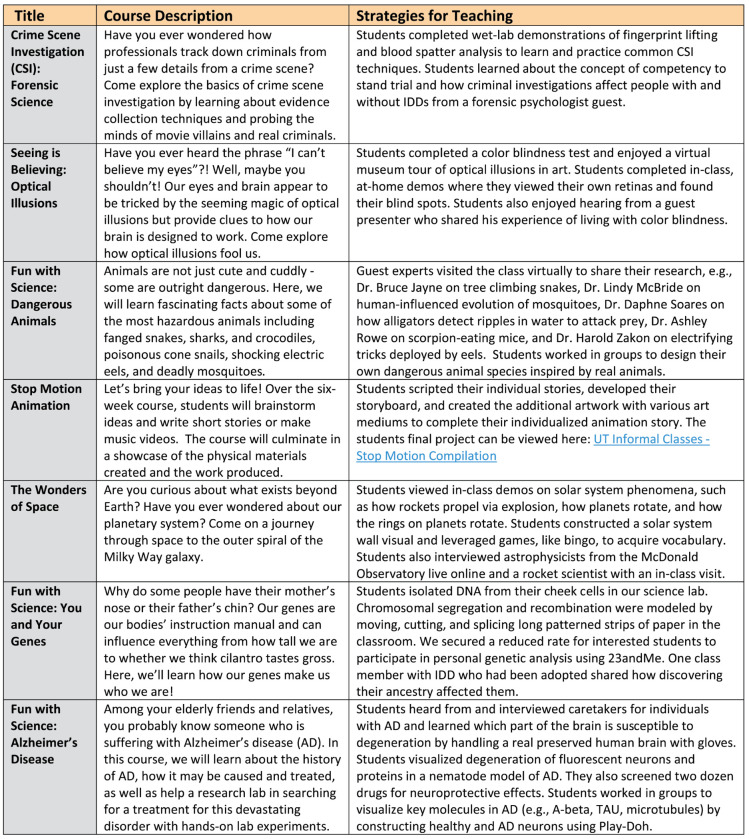
Representative Lifelong Learning with Friends science courses. Examples of 7 of the 52 past LLWF courses that exemplify the range of science courses across sophisticated academic topics. See File 1 in https://dataverse.tdl.org/dataset.xhtml?persistentId=doi:10.18738/T8/H7LOBC for more information.

LLWF is a reverse-inclusion continuing-education program aimed at adults with IDDs at the University of Texas at Austin. Reverse inclusion approaches recruit neurotypical students into special education settings to foster positive peer interaction, model appropriate behavior, and offer academic support if needed [8]. LLWF utilizes a 2:1 reverse-inclusion dynamic to allow for bidirectional social and academic learning between students with IDDs and volunteers. Adults with IDDs select courses à la carte ($125) suited to their personal interests. Each course consists of 6 classes offered once a week (Monday to Thursday evenings or Sunday afternoon) for 2 to 3 h. LLWF offers a range of courses across sophisticated academic and recreational topics that are typical of a college curriculum but are commonly unavailable to adults with IDDs [5]. Fresh course topics bolstered with new material and guest experts are intended to appeal to both adults with IDDs and volunteers (Fig 1). Universal learning design approaches help engage students with a wide range of abilities. Public visibility of our students with IDDs on university grounds can demonstrate to volunteers and others that people with IDDs belong in higher education settings. By meeting at a popular cafe on campus and holding classes in STEM buildings, LLWF is helping to break down barriers and increase awareness of IDDs in higher education.

## College volunteer recruitment and benefits

Undergraduate and graduate students were recruited through university forums, Listservs, and student organizations to participate in LLWF. Volunteers are expected to attend every class, participate in classwork and discussions, and complete homework assignments. During a mandatory orientation, volunteers learn they are expected to assume 5 roles during volunteering: mentor, peer, student, friend, and advocate. With the help of instructors, volunteers are coached on how to provide disability-competent support and to bring their new understanding of IDDs into their daily life and future careers.

**Fig 2:**
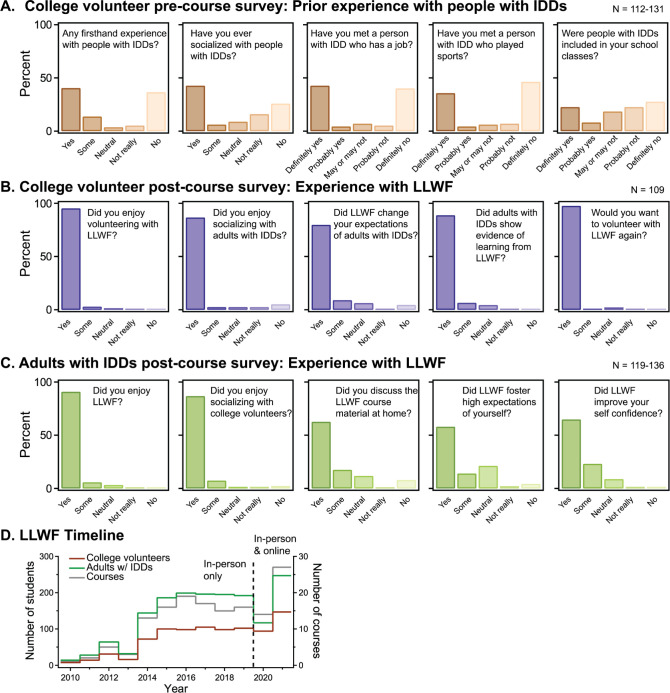
Pre- and post-course survey responses of Lifelong Learning with Friends participants and program growth. Prior to volunteering with LLWF, college students were anonymously surveyed regarding their prior experience with people with IDDs (A). In anonymous post-course surveys, volunteers were asked to rate enjoyment and convenience of LLWF as well as whether their expectations of people with IDDs changed (B). In parallel, adults with IDDs were anonymously surveyed with their family members on enjoyment of LLWF as well as potential benefits to adults with IDDs (C). LLWF enrolment and courses have grown in number since its inception in 2010 with a dip during the global COVID-19 pandemic and transition to offer both online and in-person courses (D). Note that LLWF has utilized slightly different versions of surveys, which accounts for the variation in sample sizes. See Files 2 and 3 in https://dataverse.tdl.org/dataset.xhtml?persistentId=doi:10.18738/T8/H7LOBC for more information.

We administered pre-course surveys that showed that many volunteers had little to no prior experience with adults with IDDs (Fig 2A) and post-course surveys that determined volunteers report LLWF to be a convenient and enjoyable volunteer activity that changed their expectations of people with IDDs (Fig 2B). Despite the bimodal distribution in terms of prior experience, nearly all volunteers reported that the course had changed their expectations of people with IDDs (Fig 2B). In follow-up interviews, many volunteers reported being impressed with the ability of adults with IDDs to participate meaningfully in class and to relate to them. They also reported that LLWF had positively influenced their interest in fields of study related to IDDs, such as medicine, education, or research. Most volunteers wanted to volunteer again; over 12 years, 63% of volunteers returned for subsequent semesters and 32% signed up for more than 1 course. Some students with more than a year of experience transitioned to become instructors for LLWF to teach their specialized topics (e.g., CSI and optical illusions).

In post-course surveys distributed to adults with IDDs, we determined that LLWF was considered an enjoyable program that boosted self-confidence and encouraged learning outside of class (Fig 2C). Analysis of enrollment since inception of LLWF in 2010 revealed growth in volunteers, adults with IDDs, and courses (Fig 2D).

## Factors for replication

The development of LLWF has relied on 6 factors:

1. A founder or director with the passion and drive to develop a program that provides a college-level education to adults with IDDs.
2. A sponsor for the program to take place.
3. Instructors, preferably with experience in special education or IDD services (e.g., transition specialists, art or music teachers, theater instructors, and Special Olympics coaches).
4. College students interested in volunteering as class peers.
5. Adults with IDDs and their caretakers interested in postsecondary education options.

We found that there are benefits of having a tenure-track professor initiate LLWF on our university campus. A tenured professor may have the ability to reserve classrooms on campus, such as conference rooms and science labs, that an outside instructor or non-tenure track professor may not. Professors also may more easily recruit peer professors, graduate students, and postdocs to serve as guest lecturers, which helps fortify learning. If program founders have family members with IDDs, then they may also provide a more compelling personal account to develop the program when communicating to people unfamiliar with IDDs.

Replicating our program may be particularly successful if done in collaboration with the school’s neuroscience department. We attribute much of our success in recruiting and retaining volunteers to the rapid growth of majors relevant to IDD study and service. From 2017 to 2022, neuroscience represented one of the fastest (50%) growing majors at the University of Texas. Additionally, outreach to similar IDD support organizations in Texas, such as Down Syndrome Association of Central Texas, Autism Society of Texas, Adults Independent and Motivated, and Best Buddies have helped advertise LLWF to potential students in and outside of Austin.

## Expanding online

In response to the COVID-19 pandemic, LLWF pivoted to offer virtual courses beginning in spring 2020. Due to the protective guidelines by the US Center for Disease Control in consideration of adults with IDDs, LLWF temporarily discontinued in-person courses and initiated online courses. Although switching to an online format was done out of necessity, the transition proved to be serendipitous for LLWF to expand across the USA and Canada. We found that an option for virtual postsecondary education is especially attractive to most adults with IDDs who are limited by transportation, those who are wheelchair bound, or live in smaller areas without many IDD services.

## Limitations

Families were reluctant to enroll their students with IDDs in certain course topics that they reported seemed controversial, complex, or too mature (e.g., “Psychology of Science Fiction,” “Fun with Science: Viruses,” “Fun with Cultures: World Religions,” and “Art of Frida Kahlo”). Nevertheless, we often secured better enrolment by re-marketing them with more lighthearted titles later. For example, a course originally focused on business and marketing gained better enrolment when rebranded as the more palatable “History of Walt Disney,” and a course on intimacy and consent was retitled “Romance in the Movies.” We also merged previously drier course topics, such as math and etiquette, into an enticing “It’s my party!” course where students were coached to work together to budget and plan a catered banquet in the University of Texas Tower (see [9] for more information). On occasion, we also met institutional barriers for LLWF (e.g., rooms or supplies were reserved for college students). To achieve acceptance, we reframed our requests, emphasizing that our program benefits college students by teaching them about disabilities. These problems and solutions demonstrate how a reverse-inclusion format can offer a strategy to gain wider buy-in.

## Conclusion

LLWF is an inclusive education program that provides a valuable experiential volunteering opportunity for college students, while also improving social and educational outcomes for adults with IDDs. Our results indicate that the program is successful in changing volunteers’ expectations of people with IDDs primarily through opportunities for bidirectional learning in the classroom. LLWF has reached hundreds of students with and without IDDs each year and more than 1,500 over the past 12 years (Fig 2D). The program can be replicated at other colleges to enhance inclusivity and improve societal acceptance of people with IDDs. It benefits college students by having them learn alongside students with IDDs, improves their scientific communication skills, and fosters a more inclusive society.
